# A randomized controlled trial of intranasal oxytocin in Phelan-McDermid syndrome

**DOI:** 10.1186/s13229-021-00459-1

**Published:** 2021-09-30

**Authors:** J. Fastman, J. Foss-Feig, Y. Frank, D. Halpern, H. Harony-Nicolas, C. Layton, S. Sandin, P. Siper, L. Tang, P. Trelles, J. Zweifach, J. D. Buxbaum, A. Kolevzon

**Affiliations:** 1 Icahn School of Medicine at Mount Sinai, New York, NY USA; 2 Seaver Autism Center for Research and Treatment, Icahn School of Medicine at Mount Sinai, One Gustave L. Levy Place, Box 1230, New York, NY 10029 USA; 3 Department of Psychiatry, Icahn School of Medicine at Mount Sinai, New York, NY USA; 4 Department of Neuroscience, Icahn School of Medicine at Mount Sinai, New York, NY USA; 5 Department of Pediatrics, Icahn School of Medicine at Mount Sinai, New York, NY USA; 6 Department of Medical Epidemiology and Biostatistics, Karolinska Institutet, Stockholm, Sweden; 7 Department of Genetics and Genomic Sciences, Icahn School of Medicine at Mount Sinai, New York, NY USA

**Keywords:** Phelan-McDermid syndrome, PMS, Shank3, Autism spectrum disorder, ASD, Oxytocin

## Abstract

**Background:**

Phelan-McDermid syndrome (PMS) is a rare neurodevelopmental disorder caused by haploinsufficiency of the *SHANK3* gene and characterized by global developmental delays, deficits in speech and motor function, and autism spectrum disorder (ASD). Monogenic causes of ASD such as PMS are well suited to investigations with novel therapeutics, as interventions can be targeted based on established genetic etiology. While preclinical studies have demonstrated that the neuropeptide oxytocin can reverse electrophysiological, attentional, and social recognition memory deficits in *Shank3*-deficient rats, there have been no trials in individuals with PMS. The purpose of this study is to assess the efficacy and safety of intranasal oxytocin as a treatment for the core symptoms of ASD in a cohort of children with PMS.

**Methods:**

Eighteen children aged 5–17 with PMS were enrolled. Participants were randomized to receive intranasal oxytocin or placebo (intranasal saline) and underwent treatment during a 12-week double-blind, parallel group phase, followed by a 12-week open-label extension phase during which all participants received oxytocin. Efficacy was assessed using the primary outcome of the Aberrant Behavior Checklist-Social Withdrawal (ABC-SW) subscale as well as a number of secondary outcome measures related to the core symptoms of ASD. Safety was monitored throughout the study period.

**Results:**

There was no statistically significant improvement with oxytocin as compared to placebo on the ABC-SW (Mann–Whitney *U* = 50, *p* = 0.055), or on any secondary outcome measures, during either the double-blind or open-label phases. Oxytocin was generally well tolerated, and there were no serious adverse events.

**Limitations:**

The small sample size, potential challenges with drug administration, and expectancy bias due to relying on parent reported outcome measures may all contribute to limitations in interpreting results.

**Conclusion:**

Our results suggest that intranasal oxytocin is not efficacious in improving the core symptoms of ASD in children with PMS.

*Trial registration* NCT02710084.

**Supplementary Information:**

The online version contains supplementary material available at 10.1186/s13229-021-00459-1.

## Background

Gene discovery approaches, followed by functional analysis using model systems, have clarified the neurobiology of several genetic subtypes of autism spectrum disorder (ASD) and led to important opportunities for developing novel therapeutics [44, 48, 60, 77]. ASD is now understood to have multiple distinct genetic risk loci, and one example is *SHANK3*, where haploinsufficiency through deletion or sequence variants causes Phelan-McDermid syndrome (PMS), which is characterized by global developmental delay, motor skills deficits, delayed or absent speech, and ASD [71]. *SHANK3* is the critical gene in this syndrome [15, 16, 29], and studies indicate that loss of one copy of *SHANK3* causes a monogenic form of ASD with a frequency of at least 0.5% of ASD cases and up to 2% of ASD with moderate to profound intellectual disability [53]. * SHANK3* codes for a master scaffolding protein in postsynaptic glutamatergic synapses and plays a critical role in synaptic function [14]. Using *Shank3*-deficient mice and rats, specific deficits in synaptic function and plasticity in glutamate signaling have been identified [17, 42, 44, 45, 47, 59, 70, 81]. Importantly, studies in *Shank3*-deficient rats have demonstrated that oxytocin reverses synaptic plasticity deficits in the hippocampus and the medial prefrontal cortex, in addition to reversing behavioral deficits in long-term social recognition memory and attention [45]. Furthermore, oxytocin has recently been shown to stimulate neurite outgrowth and increase gene expression of Shank3 protein in human neuroblastoma cells [84] and to reverse neurite abnormalities in *Shank3-*deficient mice [65].

Oxytocin is an FDA-approved, commercially available medication that can be compounded into an intranasal solution for passage through the blood–brain barrier (BBB). Oxytocin is the brain’s most abundant neuropeptide; it can act as a classical neurotransmitter, a neuromodulator, and a hormone with actions throughout the body [10, 36, 78]. In animal models, oxytocin has been demonstrated to increase social approach behavior, social recognition, social memory, and to reduce stress responses [18, 21, 52]. In humans, oxytocin is known as a strong modulator of social behavior and increases gaze to eye regions, social cognition, social memory, empathy, perceptions of trustworthiness, and cooperation within one’s own group [9, 11, 22–25, 28, 34, 35, 38, 39, 49–51, 58, 63, 66, 67, 68, 75, 76, 83].

Studies of intranasal oxytocin suggest equivocal effects on social behavior both generally and in ASD [5, 6, 22, 24–27, 40, 41, 62, 74, 82, 83]. The largest study in ASD to date did not demonstrate a significant improvement in social behavior with intranasal oxytocin [69]. There are many proposed reasons why oxytocin has proven unreliable in ASD, including due to issues around brain penetrance, dosing, and trial design [1, 54, 61]. However, the clinical and etiological heterogeneity of ASD pose prominent obstacles for successful clinical trials in ASD in general and likely contributes to challenges detecting consistent effects with oxytocin. While it is necessary to exercise caution in interpreting the literature to date as justification for the current trial, we sought to address this issue of heterogeneity by recruiting a sample population with a shared underlying genetic etiology. The following study was conducted to assess the safety and efficacy of oxytocin in children with PMS, all of whom had a deletion or pathogenic sequence variant of the *SHANK3* gene. We hypothesized that individuals with PMS would show improvement in social withdrawal symptoms following oxytocin administration.

## Methods

We used a double-blind, placebo-controlled parallel group design in 18 children with PMS, aged 5 to 17 years old, to evaluate the impact of oxytocin on impairments in socialization, language, and repetitive behaviors. The study protocol was approved by the Mount Sinai Program for the Protection of Human Subjects, and all caregivers signed informed consent. Participants were enrolled between May 2016 and November 2019 and randomized to receive either intranasal oxytocin or matching placebo (intranasal saline) for 12 weeks during the double-blind phase, followed by a 12-week open-label extension with intranasal oxytocin.

All participants had pathogenic deletions encompassing *SHANK3* (*n* = 12) or *SHANK3* sequence variants (*n* = 6). Among participants with deletions, two had ring chromosome 22. The mean deletion size was 3.1 Mb (range = 55 Kb–8.2 Mb; SD = 2.7 Mb). Among participants with sequence variants, three had p.Ala1227Glyfs*69, two had p.Leu1142Valfs*153, and one had p.Ile1094Thrfs*100. Seventeen of 18 participants met criteria for ASD based on clinical consensus using the Autism Diagnostic Observation Schedule, Second Edition (ADOS-2) [56], the Autism Diagnostic Interview-Revised (ADI-R) [55], and the Diagnostic Manual for Mental Disorders, Fifth Edition (DSM-5; [4]).

The primary outcome measure was the Aberrant Behavior Checklist-Social Withdrawal subscale (ABC-SW; [2]) and was selected to capture a core symptom domain in ASD. Secondary outcome measures included the Repetitive Behavior Scale-Revised (RBS-R, [13]), the Short Sensory Profile (SSP, [30]), the Macarthur-Bates Communicative Development Inventory (MCDI, [32, 33]), the Vineland Adaptive Behavior Scales, Second Edition [73], the Clinical Global Impression-Improvement Scales (CGI-I, [43]), and the Mullen Scales of Early Learning (MSEL; [57]). The MSEL was chosen to assess cognitive ability as recommended in individuals with PMS who are often unable to complete standardized IQ tests [72], developmental quotients were calculated as previously described in the literature [31] to evaluate baseline ability, and age equivalents were used to assess change with treatment.

Additional exploratory measures were administered using electrophysiology and eye tracking paradigms, and if feasibility is established, results will be reported in a subsequent publication.

To be eligible to participate, all participants had a minimum raw score of 12 on the ABC-SW at enrollment, which was selected by adding approximately one standard deviation to the mean ABC-SW subscale score derived from a normative sample of 601 children aged 6–17 with intellectual disability [19] and as suggested by Aman and Singh [3]. All participants were on stable medication regimens for at least three months prior to enrollment and throughout the study period.

### Drug administration

Oxytocin was delivered in 5-ml bottles with a dosage pump manufactured by Novartis in Europe and marketed as Syntocinon™ under an Investigational New Drug Application (IND) from the FDA (IND #104496). The first seven participants started the trial with a dose of 24 international units (IU) twice daily (BID), which was adjusted to 12 IU BID after two participants experienced increased irritability. Subsequently, after the first two-week check-in call, if the drug was well tolerated, the dose was increased to 24 IU BID. Each insufflation delivered 4 IU and three insufflations (12 IU) in each nostril were given twice daily for a total daily dose of 48 IU. The dose of 24 IU BID was chosen because it is the most commonly used in the literature in sample populations with ASD [5, 6, 40, 41, 62, 74]. Identical doses were used during the open-label treatment phase. Adherence to treatment was assessed by caregiver report and drug diary. Randomization and blinding was performed by the Mount Sinai Research Pharmacy. Caregivers received written instructions for how to administer the study drug, and the first dose was administered by the principal investigator with the caregiver observing.

Efficacy and safety measurements were taken at baseline, and at weeks 4, 8, and 12 of each treatment phase (double blind and open-label). An additional safety assessment was done at week 2 in both phases. Monitoring of adverse events (AEs) was done using an adapted semi-structured interview, the Safety and Monitoring Uniform Report Form (SMURF). AEs were documented with respect to severity, duration, management, relationship to study drug, and outcome. Severity was graded using a scale of mild, moderate, or severe. Primary and secondary outcomes were administered by an independent evaluator (DH), who was blind to side effects to prevent the risk of bias.

### Data analysis

All statistical computing was performed in SPSS Version 27. Analyses were performed on the intent-to-treat population. All data were explored for outliers and data quality using descriptive statistics and graphs prior to breaking of the blind and any statistical hypothesis testing. For data on a categorical scale, we used contingency tables and histograms, and for data on a continuous scale, box-and-whisker plots.

We tested for differences in change from baseline to week 12 of the primary efficacy variable, ABC-SW, as well as all other variables by calculating the Mann–Whitney U test [46]. The Mann–Whitney U test is a nonparametric test robust to single gross outliers and does not require the data to follow any particular data distribution. We compared within subject changes from week 12 to 24 by calculating the Wilcoxon signed rank test [80]. In supplementary analyses, we fitted generalized linear regression models assuming data to follow an approximate normal distribution, which allowed us to include and adjust for the baseline value. All tests of statistical hypotheses were done on the two-sided 5% level of significance and were completed while remaining blind to treatment assignments. After selecting a single primary efficacy variable, we did not adjust for multiplicity of statistical tests. However, all raw *p*-values are provided for any post hoc adjustment.

Missing data

In the case of two participants who dropped out after week 4, difference scores were calculated for each outcome measure using the last observation carried forward. Two participants dropped out after baseline and were not included in the efficacy analysis (see Safety section).

## Results

### Double-Blind phase: baseline to week 12

**Fig. 1:**
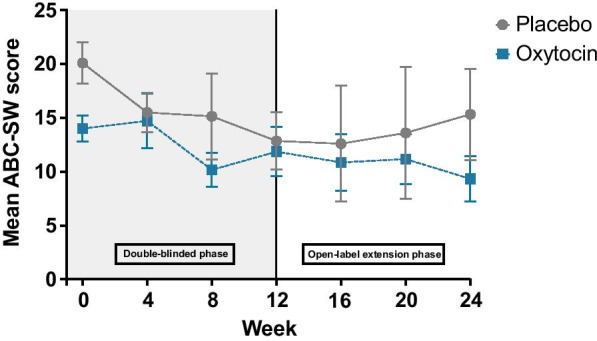
Mean scores on the Aberrant Behavior Checklist Social Withdrawal subscale across study visits. Bars represent standard error

Baseline characteristics were similar between groups, with the exception of ABC-SW scores (Table 1). Sixteen participants were included in the efficacy analysis (See CONSORT diagram; Additional file 2: figure 2). There was no statistically significant difference between oxytocin and placebo groups on change from baseline to week 12 on the ABC-SW, our primary outcome (*U* = 50, median placebo change =  − 7; median oxytocin change =  − 3; *p* = 0.055) (Fig. 1). This result did not change in a regression analysis controlling for baseline differences in ABC-SW score (*F* = 3.41, *p* = 0.088) or after removing the two participants who dropped out after week 4 (*U* = 39.5, *p* = 0.053). In addition, there were no statistically significant differences between groups for any of our secondary outcomes during the double-blind phase (Table 2).

**Table 1:** Participant baseline characteristics

Sex	Placebo (N = 10) *N* F: 5 M: 5		Range	Oxytocin (N = 8) *N* F: 4 M: 4	Range	Total (N = 18) *N* F: 9 M: 9	Range	*p*-value
	Mean (SD)			Mean (SD)		Mean (SD)		
Age (years)	9.8 (3.6)		6–17	6.8 (1.4)	5–9	8.4 (3.2)	5–17	0.055
Baseline ABC-SW	20.1 (6.1)		12–30	14 (3.4)	12–20	17.4 (5.9)	12–30	0.012*
Verbal DQ	14.9 (12.8)		4–46	26.4 (21.3)	8–61	20 (17.6)	4–61	0.203
Nonverbal DQ	20.1 (11.7)		4–42	26.1 (13.1)	11–50	22.7 (12.3)	4–50	0.408
Full Scale DQ	17.4 (11.8)		5–44	26.3 (16.7)	10–50	21.4 (14.5)	5–50	0.173

*ABC-SW* Aberrant Behavior Checklist Social Withdrawal subscale; *DQ* developmental quotient; *F* female; *M* male; *N* sample size; *SD* standard deviation

**Table 2:** Comparison across groups of mean change from baseline to week 12

Measure	Variable name	Number of subjects placebo	Mean change placebo	Median change placebo	Number of subjects oxytocin	Mean change oxytocin	Median change oxytocin	Mann–Whitney U test statistic	Two-sided p-value
ABC									
	Irritability	9	− 5	− 3	7	− 1.71	0	40.5	0.351
	Social withdrawal	9	− 7.44	− 7	7	− 2.42	− 3	50	0.055
	Stereotypy	9	− 2.22	− 1	7	0.57	0	41.5	0.299
	Hyperactivity	9	− 8.11	− 6	7	− 2.71	0	42	0.299
	Inappropriate speech	9	− 1.67	0	7	− 7.14	0	35	0.758
RBS-R									
	Stereotypic behaviors	9	− 1.67	− 2	7	0.29	0	43	0.252
	Self-injury	9	− 1.22	0	7	1	0	46	0.142
	Compulsive behaviors	9	− 1.56	0	7	− 0.14	0	38	0.536
	Ritualistic behaviors	9	− 1.33	− 1	7	− 0.43	0	39	0.47
	Sameness behaviors	9	− 2	− 1	7	− 0.29	− 1	41	0.351
	Restricted behaviors	9	− 1.43	0	7	0.14	0	23.5	0.902
	Overall score	9	− 8.56	− 3	7	0	0	46	0.142
CGI-I									
	Severity	9	− 0.11	0	7	− 0.14	0	30.5	0.918
	Improvement*	9	N/A	N/A	7	N/A	N/A	38	0.536
SSP									
	Tactile	9	2.56	2	7	1.57	0	29	0.837
	Taste/smell	9	1.67	0	7	0.71	0	28.5	0.758
	Movement	9	− 0.67	0	7	0.29	0	41.5	0.299
	Under-responsive/seeks sensation	9	4.44	6	7	0.71	2	19	0.21
	Auditory filtering	9	4	3	7	0.29	− 1	16	0.114
	Low energy/weak	9	1.67	2	7	− 0.71	0	24	0.47
	Visual/auditory sensitivity	9	1.89	3	7	− 0.86	0	13	0.055
	Total	9	14.33	8	7	1	− 8	14.5	0.071
Vineland-II									
	Communication	7	4	0	6	2.83	2	22	1
	Daily living skills	7	3.29	1	6	1.17	0.5	14.5	0.366
	Socialization	7	3	1	6	− 0.5	− 0.5	12	0.234
	Motor	6	1	0	6	3.17	0	20.5	0.699
	Adaptive behavior composite	7	3.14	2	6	2	2	21	1
	Internalizing	7	− 1.14	− 1	6	− 0.5	0	29	0.295
	Externalizing	7	− 1.14	− 1	6	− 0.17	0	25.5	0.534
	Maladaptive	6	− 1.33	0	6	− 0.5	− 0.5	18	1
MSEL									
	Gross motor	7	− 1.57	0	7	− 0.86	0	22	0.805
	Visual reception	7	0.29	0	7	2.86	4	34	0.259
	Fine motor	7	− 0.29	0	7	0.71	0	32	0.383
	Receptive language	7	− 1.86	− 1	7	1.71	1	35.5	0.165
	Expressive language	7	0.43	0	7	0.43	0	22	0.805
MCDI									
	Phrases understood	7	0.71	0	7	1.43	0	29.5	0.535
	Words understood	7	− 8.14	0	7	7	1	29	0.62
	Words produced	7	− 8.43	0	7	2.57	0	23	0.902
	Early gestures	7	1.86	1	7	− 0.27	0	9	0.053
	Later gestures	7	2.71	1	7	0.86	1	26	0.902
	Total gestures	7	4.57	1	7	0.57	1	18	0.456

^*^CGI-I scores reflect results at Week 12 only; Vineland-II domain and composite values are standard scores; MSEL values are age equivalents

*ABC* Aberrant Behavior Checklist; *AE* age equivalent; *CGI* Clinical Global Impression-Improvement Scale; *MCDI* Macarthur-Bates Communicative Development Inventory; *MSEL* Mullen Scales of Early Learning; *RBS* Repetitive Behavior Scale-Revised; *SSP* Short Sensory Profile

Five participants were receiving concomitant psychotropic medications during the study period; three were randomized to the placebo group and two were randomized to oxytocin. All doses remained stable throughout the trial. Among participants in the placebo group, psychotropic medications included aripiprazole, tizanidine, mirtazapine, and doxepin (*n* = 1), clonidine and lisdexamfetamine (*n* = 1), divalproex sodium (*n* = 1), and melatonin (*n* = 2). Among the participants in the oxytocin group, medications included gabapentin, oxcarbazepine, and melatonin (*n* = 1) and levetiracetam (*n* = 1).

### Open-label phase: week 12 to week 24

We further examined within-subject change on all outcome measures during the open-label phase of the trial for each group individually and across all participants. There were no statistically significant improvements on any outcome measure between week 12 and week 24 in either group or across all participants.

### Safety

Eighteen participants were included in the safety analysis. Oxytocin was generally well tolerated, and there was no statistically significant difference between groups in the frequency of AEs (*U* = 37, *p* = 0.83). Three participants withdrew from the study during the double-blind phase due to tolerability concerns: one participant developed croup and a sinus infection (placebo) and the caregivers stopped the study drug on their own; two participants developed worsening irritability (one placebo; one oxytocin) and withdrew in consultation with the principal investigator. A fourth participant dropped out after receiving a single dose of study drug (placebo) due to concerns unrelated to tolerability (Additional file 2: figure 2). There were no serious adverse events (Table 3).

**Table 3:** Reported adverse events from randomization through week-12

Adverse events	Placebo	Oxytocin
Sedation	2 (20%)	1 (12.5%)
Decreased appetite	2 (20%)	0 (0%)
Periorbital/facial swelling	1 (10%)	0 (0%)
Diarrhea	1 (10%)	1 (12.5%)
Upper respiratory tract infection	2 (20%)	1 (12.5%)
Sleep disturbance	2 (20%)	5 (62.5%)
Increased appetite	1 (10%)	1 (12.5%)
Irritability/agitation	3 (30%)	1 (12.5%)
Cough	0 (0%)	1 (12.5%)
Runny nose/congestion	1 (10%)	0 (0%)
Fever	5 (50%)	2 (25%)
Aggression/self-injury	1 (10%)	0 (0%)
Infection	3 (30%)	2 (25%)
Elated mood/silliness	1 (10%)	3 (37.5%)
Leg weakness	1 (10%)	1 (12.5%)
Restlessness/hyperactivity	1 (10%)	5 (62.5%)
Bloody nose	0 (0%)	2 (25%)
Stereotypies	2 (20%)	1 (12.5%)
Apathy	0 (0%)	1 (12.5%)
Foot pain	1 (10%)	0 (0%)
Hirsutism	0 (0%)	1 (12.5%)
Tooth pain	0 (0%)	1 (12.5%)
Eczema	1 (10%)	1 (12.5%)
Allergies/asthma	2 (20%)	2 (25%)
Enuresis	1 (10%)	0 (0%)
Accidental injury	1 (10%)	0 (0%)
Seizure	1 (10%)	0 (0%)
Rubbing ears	1 (10%)	0 (0%)
Disinhibited	1 (10%)	0 (0%)
Oppositional behavior	2 (20%)	0 (0%)
Low frustration tolerance	1 (10%)	1 (12.5%)
Tantrums	0 (0%)	1 (12.5%)
Total	41	35

## Discussion

The results of this small study do not support the use of intranasal oxytocin in PMS. Despite promising preclinical data and a plausible biological rationale, attempts to reduce genetic heterogeneity by selecting only participants with PMS were not adequate to identify a population in which oxytocin treatment would show more uniform impact on social behavior. The lack of a treatment effect highlights the challenges in translating from animal and other preclinical models to humans. While preclinical models may show strong construct validity, outcome measures, dosing, and bioavailability are difficult to replicate in humans. Outcome measures in preclinical studies have the advantage of enhanced objectivity and quantifying change, while clinical studies in ASD rely mainly on parent-report measures which capture ratings of behavior using relatively broad questions and are vulnerable to subjective bias. Further, while most of the outcome measures used in this study, including the ABC-SW, have been carefully validated in ASD and intellectual disability, none have undergone rigorous psychometric testing in PMS as of yet.

In terms of dosing, most studies with intranasal oxytocin in ASD continue to use 24 IU, but there have been no dose response studies to establish the appropriate dosing for different ages or populations. Differential effects may also occur based on acute versus chronic dosing; both methods appear to enhance functional connectivity in adult wild-type mice, but repeated administration was associated with reduced social interaction and communication in at least one study [61]. Likewise, differential effects were evident in a recent clinical trial in adults with ASD using proton magnetic resonance spectroscopy such that chronic dosing was associated with significant reductions in medial prefrontal cortical *N*-acetylaspartate and glutamate levels, whereas acute dosing was not [12]. Equally important, intranasal delivery is fraught with challenges, especially for severely impaired populations who do not reliably follow instructions. Several studies have established brain penetrance with intranasal oxytocin in ASD based on functional imaging studies, [7, 8, 26, 37, 79], but participants were mostly adult males without comorbid intellectual disability. As such, differential effects across studies and sample populations may well relate to poorly controlled delivery and variability in absorption from the olfactory epithelium [1, 64]. However, this variability does not appear to contribute to tolerability issues, and AEs did not differ between groups in the present study, a finding consistent with at least one large meta-analysis of reported AEs from clinical trials with intranasal oxytocin in ASD [20]. Also, worth noting, in the study with *Shank3-*deficient rats that served in part as the basis for this clinical trial, oxytocin was administered though intracerebroventricular injection and not intranasally [45]. Future studies might also control for baseline levels of oxytocin as variability in underlying oxytocin regulation may contribute to differential responses; individuals with lower pre-treatment oxytocin levels may show the greatest improvement in social responsiveness [62].

### Limitations

Drawing definitive conclusions about the efficacy of oxytocin in PMS based on the results of this study is limited by our small sample size, a challenge inherent to studying rare disorders. In fact, we ended our trial after three years due to challenges with recruitment and perhaps stopped before enrolling an adequate sample size to detect small effects. Our dosing paradigm also did not account for the relatively broad age range, pubertal status, or baseline levels of oxytocin. In addition, despite adequate training on drug administration, six insufflations twice daily may have been challenging for some participants. Compliance was monitored using parent report and drug diaries, but given that our study drug was administered at home, it is possible that doses were missed or not properly administered. Further, the majority of our measures, including the primary outcome, rely on subjective parent reporting and have not been well validated for use in clinical trials in PMS. Finally, preclinical models benefit from genetic homogeneity and while all the individuals in our sample had *SHANK3* haploinsufficiency, it was comprised of those with 22q13 deletions of varying sizes (*n* = 12), in addition to *SHANK3* sequence variants (*n* = 6). However, the present study was too small to examine subset analyses but this may be a worthy endeavor for future studies.

## Conclusions

While the results of this study must be interpreted with caution in light of the limitations, intranasal oxytocin does not appear efficacious in improving the core symptoms of ASD in children with PMS. Despite these findings and inconsistent results across studies, enthusiasm persists for oxytocin as a treatment for ASD symptoms. Future studies must address this complex multitude of confounding factors to advance oxytocin as a treatment in ASD and related neurodevelopmental disorders. In addition to genetics, other biomarkers, such as using electrophysiology, to stratify sample populations and attempt to predict treatment response have the potential to improve clinical trial designs. The development of new outcome measures, refined and validated for specific genetic forms of ASD, may also help. Importantly, outcome measures that incorporate objective assessments will be more likely to reduce bias. Finally, exploring novel mechanisms for intranasal delivery to enhance absorption in addition to clarifying optimal dosing regimens will be critical for future trial success. Nevertheless, a genetics-first approach and other methods to limit heterogeneity in clinical trial populations remain a promising path forward for studies in ASD and related neurodevelopmental disorders.

## Supplementary Information

**Additional file 1:Supplementary Table 1:** Mean values for all measures across groups at baseline and week 12.

**Additional file 2: figure 2**. CONSORT diagram showing the flow of participants through Week 12

## Data Availability

The datasets used and/or analyzed during the current study are available from the corresponding author on reasonable request.

## Abbreviations

ABC: Aberrant Behavior Checklist
ADI-R: Autism Diagnostic Interview-Revised
ADOS-2: Autism Diagnostic Observation Schedule, Second Edition
DSM-5: Diagnostic and Statistical Manual for Mental Disorders, Fifth Edition
AE: Age equivalent
AEs: Adverse events
ASD: Autism spectrum disorder
DQ: Developmental quotient
CGI-I: Clinical Global Impression-Improvement Scale
FDA: Food and Drug Administration
IU: International units
MCDI: Macarthur-Bates Communicative Development Inventory
MSEL: Mullen Scales of Early Learning
PMS: Phelan-McDermid syndrome
RBS-R: Repetitive Behavior Scale-Revised
SMURF: Safety monitoring report form
SSP: Short Sensory Profile
Vineland-II: Vineland Adaptive Behavior Scales
