# Supplementary material for: A randomized controlled trial of intranasal oxytocin in Phelan-McDermid syndrome
Source: Mol Autism. 2021 Sep 30;12:62. doi: 10.1186/s13229-021-00459-1 (PMC8482590; doi:10.1186/s13229-021-00459-1)
Supplement: Supplementary file 1 — Additional file 1:Supplementary Table 1: Mean values for all measures across groups at baseline and week 12. [file 13229_2021_459_MOESM1_ESM.docx]

**Supplement**

***Supplementary Table 1: Mean values for all measures across groups at baseline and week 12***

| **Measure** | **Variable name** | **Number of Subjects Placebo** | **Baseline Mean (SD) Placebo** | **Week 12 Mean Placebo** | **Number of Subjects Oxytocin** | **Baseline Mean (SD) Oxytocin** | | **Week 12 Mean Oxytocin** |
| --- | --- | --- | --- | --- | --- | --- | --- | --- |
|  |  |  |  |  |  |  |  |  |
| **ABC** |  |  |  |  |  |  | |  |
|  | Social withdrawal | 9 | 19.67 (6.34) | 12.22 (6.24) | 7 | 14.29 (3.59) | | 11.86 (6.04) |
|  | Irritability | 9 | 10.33 (5.24) | 5.33 (3.12) | 7 | 9.57 (8.75) | | 7.86 (8.31) |
|  | Stereotypy | 9 | 9.22 (4.24) | 7 (4.8) | 7 | 6.29 (3.04) | | 6.86 (2.48) |
|  | Hyperactivity | 9 | 29.67 (9.39) | 21.56 (12.42) | 7 | 21.71 (12.88) | | 19 (10.74) |
|  | Inappropriate speech | 9 | 4 (3.64) | 2.33 (3.16) | 7 | 3 (4.04) | | 2.29 (2.36) |
| **RBS-R** |  |  |  |  |  |  | |  |
|  | Stereotypic behaviors | 9 | 5.89 (5.09) | 4.22 (3.42) | 7 | 3.57 (3.55) | | 3.86 (3.02) |
|  | Self-injury | 9 | 3.33 (4) | 2.11 (3.22) | 7 | 1.86 (2.48) | | 2.86 (4.6) |
|  | Compulsive behaviors | 9 | 4.33 (5.5) | 2.78 (4.52) | 7 | 1.29 (2.21) | | 1.14 (2.19) |
|  | Ritualistic behaviors | 9 | 3.89 (4.62) | 2.56 (3.54) | 7 | 1.86 (2.61) | | 1.43 (1.51) |
|  | Sameness behaviors | 9 | 6.33 (8.46) | 4.33 (7.81) | 7 | 3.29 (1.6) | | 3 (1.91) |
|  | Restrictive behaviors | 9 | 2.67 (2.74) | 1.89 (1.9) | 7 | 3.29 (3.55) | | 3.43 (3.41) |
|  | Overall score | 9 | 26.44 (25.55) | 18.44 (21.87) | 7 | 15.29 (10.44) | | 15.71 (12.75) |
| **CGI-I** |  |  |  |  |  |  | |  |
|  | Severity | 9 | 5 (0.5) | 5 (0.5) | 7 | 4.88 (0.354) | | 4.71 (0.488) |
|  | Improvement * | 9 | N/A | N/A | 7 | N/A | | N/A |
| **SSP** |  |  |  |  |  |  | |  |
|  | Tactile | 9 | 27 (4.06) | 29.56 (3.32) | 7 | 30.14 (4.3) | | 31.71 (2.29) |
|  | Taste/smell | 9 | 16.78 (5.86) | 18.44 (6.25) | 7 | 18.86 (3.02) | | 19.57 (1.13) |
|  | Movement | 9 | 13.56 (2.6) | 12.89 (3.44) | 7 | 14.14 (1.46) | | 14.43 (1.13) |
|  | Under-responsive/seeks attention | 9 | 16.11 (6.15) | 20.56 (8.41) | 7 | 22.43 (5.62) | | 22.71 (5.06) |
|  | Auditory filtering | 9 | 15.11 (4.97) | 19.11 (4.97) | 7 | 20.71 (4.75) | | 20 (3.11) |
|  | Low energy/weak | 9 | 19.22 (7.74) | 20.89 (8.39) | 7 | 17.43 (2.88) | | 17.71 (4.61) |
|  | Visual/auditory sensitivity | 9 | 20.11 (2.52) | 22 (2.55) | 7 | 22.29 (2.81) | | 21.43 (4.54) |
|  | Summary total | 9 | 127.89 (18.81) | 142.22 (22.15) | 7 | 146 (14.45) | | 147 (15.4) |
| **Vineland-II** |  |  |  |  |  |  | |  |
|  | Communication | 7 | 39.67 (10.79) | 43.86 (12.56) | 6 | 52.57 (14.6) | | 52.17 (10.21) |
|  | Daily Living Skills | 7 | 43.89 (9.239) | 47 (16.24) | 6 | 52.43 (12.12) | | 53 (8.51) |
|  | Socialization | 7 | 48.89 (9.26) | 50.71 (13.29) | 6 | 58 (9.64) | | 54.17 (3.92) |
|  | Motor | 6 | 57.89 (5.33) | 60.17 (8.06) | 6 | 55.14 (9.3) | | 56.33 (7.99) |
|  | Adaptive Behavior Composite | 7 | 43.22 (9.56) | 45.71 (14.02) | 6 | 54.14 (11.38) | | 53.17 (7.08) |
|  | Internalizing | 7 | 19.67 (2.65) | 19.57 (2.3) | 6 | 18.57 (2.99) | | 18.33 (2.94) |
|  | Externalizing | 7 | 16.89 (2.21) | 15.71 (1.5) | 6 | 15.57 (2.7) | | 15.17 (2.32) |
|  | Maladaptive | 6 | 19.75 (1.49) | 18.86 (2.12) | 6 | 18.57 (2.07) | | 18.33 (1.75) |
| **MSEL** |  |  |  |  |  |  | |  |
|  | Gross Motor | 7 | 30.57 (4.61) | 29 (5.29) | 7 | 22.14 (7.84) | | 21.29 (8.42) |
|  | Visual Reception | 7 | 24.71 (18.13) | 25 (20.05) | 7 | 21.57 (15.9) | | 24.43 (16.29) |
|  | Fine Motor | 7 | 21.57 (13.67) | 21.29 (12.97) | 7 | 22.14 (13.37) |  | 22.86 (14.05) |
|  | Receptive Language | 7 | 23.14 (19.68) | 21.29 (17.77) | 7 | 21.14 (19.08) | | 22.86 (19.76) |
|  | Expressive Language | 7 | 18.14 (14.83) | 18.57 (16.32) | 7 | 18.71 (20.35) | | 19.14 (21.51) |
| **MCDI** |  |  |  |  |  |  | |  |
|  | Phrases Understood | 7 | 16.29 (10.95) | 17 (11.33) | 7 | 14.57 (12.19) | | 16 (12.48) |
|  | Words understood | 7 | 198.14 (177.9) | 190 (173.57) | 7 | 166.71 (188.39) | | 173.71 (193.07) |
|  | Words produced | 7 | 106.43 (181.42) | 98 (163.28) | 7 | 146.71 (185.1) |  | 149.29 (187.72) |
|  | Early gestures | 7 | 5.29 (4.68) | 7.14 (5.27) | 7 | 8.86 (6.18) |  | 8.57 (5.94) |
|  | Later gestures | 7 | 13.14 (12.72) | 15.86 (16.54) | 7 | 11.43 (16.13) | | 12.29 (15.56) |
|  | Total gestures | 7 | 18.43 (16.99) | 23 (21.46) | 7 | 20.29 (21.71) | | 20.86 (20.75) |

*CGI-I scores reflect results at Week 12; only Vineland-II domain and composite values are standard scores; MSEL values are age equivalents.

ABC = Aberrant Behavior Checklist; AE = age equivalent; CGI = Clinical Global Impression - Improvement Scale; MCDI = Macarthur-Bates Communicative Development Inventory; MSEL = Mullen Scales of Early Learning; RBS = Repetitive Behavior Scale-Revised; SSP = Short Sensory Profile
