# Supplementary figures and images for: A randomized controlled trial of intranasal oxytocin in Phelan-McDermid syndrome
Source: Mol Autism. 2021 Sep 30;12:62. doi: 10.1186/s13229-021-00459-1 (PMC8482590; doi:10.1186/s13229-021-00459-1)

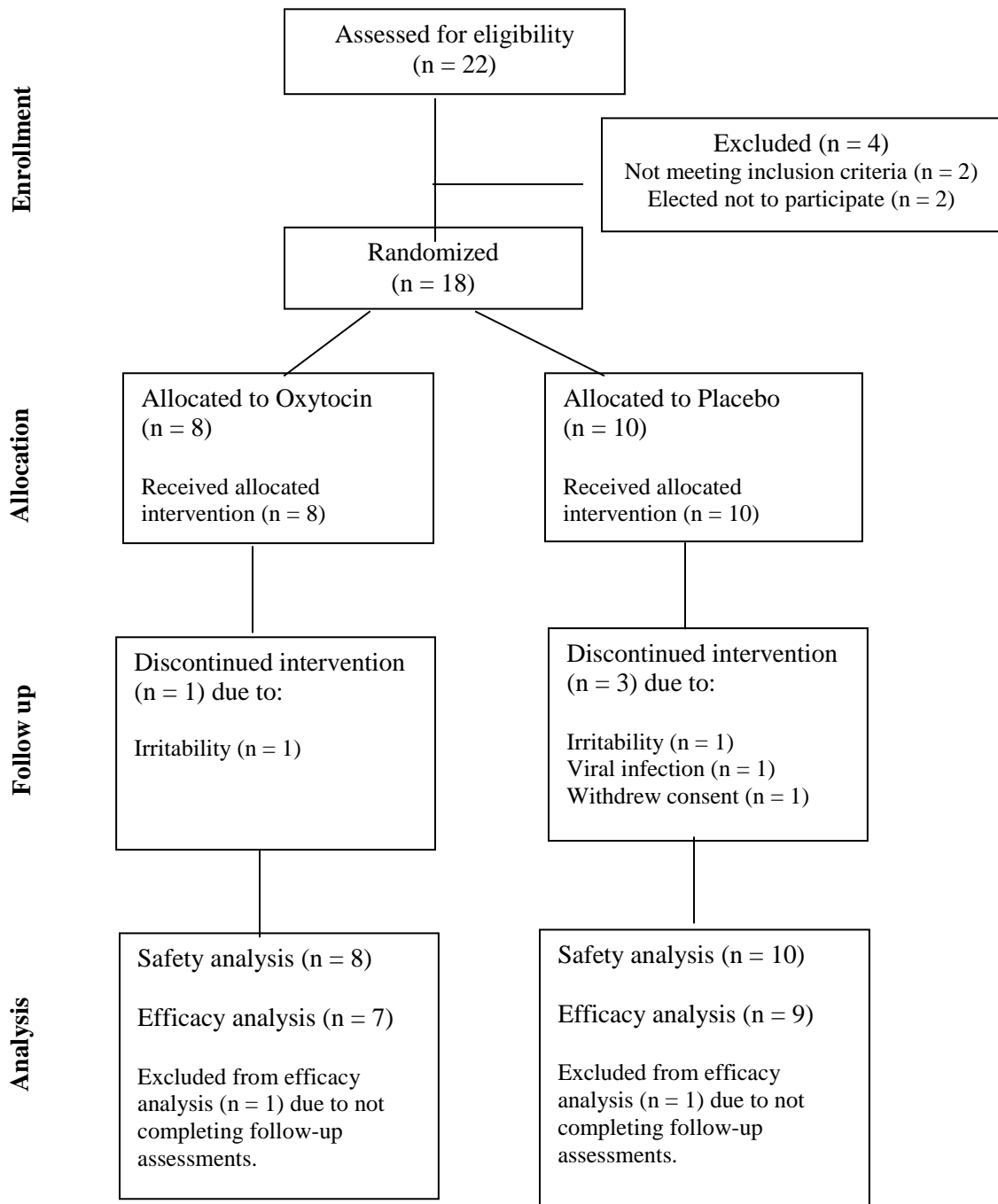

Supplement: Supplementary file 2 — Additional file 2: figure 2. CONSORT diagram showing the flow of participants through Week 12 [file 13229_2021_459_MOESM2_ESM.pdf]
